# The diagnostic value of serum miR-21 in patients with ovarian cancer: a systematic review and meta-analysis

**DOI:** 10.1186/s13048-022-00985-3

**Published:** 2022-05-02

**Authors:** Lili Qiu, Guangping Weng

**Affiliations:** Department of Laboratory, The Second Affiliated Hospital of Hainan Medical University, Haikou, 570311 Hainan China

**Keywords:** Ovarian Cancer, MiR-21, Diagnostic value, Meta-analysis

## Abstract

**Objective:**

There have been a variety of published studies on the expression of serum miR-21 in patients with ovarian cancer associated with the diagnostic value of ovarian cancer, but the conclusions are not clearly elucidated. This study aims to evaluate the value of serum miR-21 expression in the diagnosis of patients with ovarian cancer by meta-analysis.

**Methods:**

Databases, such as PubMed, Embase, Web of Science, Cochrane Library, China National Knowledge Infrastructure (CNKI), and China WanFang, were searched for relevant studies upon the correlation between the expression of serum miR-21 and the diagnostic value of ovarian cancer from inception to March 7, 2022. Statistical analysis was performed using Stata 15.0 software. The pooled sensitivity, specificity, diagnostic odds ratio (DOR), positive likelihood ratio (PLR), and negative likelihood ratio (NLR) were calculated. The meta-regression analysis and subgroup analysis were used to explore the sources of heterogeneity. The Quality Assessment of Diagnostic Accuracy Studies-2 (QUADAS-2) system was used to evaluate the quality of the included literature.

**Results:**

A total of 6 articles were included in the meta-analysis. The results showed that the pooled sensitivity, specificity, PLR, NLR, and DOR were 0.81 (95%CI: 0.73–0.88), 0.82 (95%CI: 0.75–0.87), 4.51 (95%CI: 3.22–6.31), 0.23 (95%CI: 0.16–0.33), and 19.87 (95% CI: 11.27–35.03), respectively. The area under the summary receiver operating characteristic curve was 0.89 (95%CI: 0.85–0.91). No significant publication bias was found (*P* > 0.05).

**Conclusion:**

Serum miR-21 has a good diagnostic value for ovarian cancer, which can be an ideal diagnostic biomarker for ovarian cancer. However, we should gingerly use miR-21 as a diagnostic reference standard, due to the limited number of included studies and heterogeneity.

## Introduction

Ovarian cancer is a malignant tumor of the female reproductive system, which is characterized by its insidious onset and rapid progress, among which epithelial ovarian cancer (EOC) accounts for about 90%. According to the cancer statistics, 70–80% of ovarian cancer patients have been in the advanced stage before diagnosis, and the 5-year survival rate is only 20–30%, while the 5-year survival rate for initial-stage ovarian cancer is as high as 90% [1]. Hence, improving the early diagnosis rate of ovarian cancer is beneficial in the optimal timing of treatment and obtaining a better prognosis. However, at present, there is still a lack of specific molecular markers for the diagnosis of ovarian cancer. Carbohydrate antigen 125 (CA125) is the serum marker most widely applied for ovarian cancer. Yet, in recent years, it has been found that there is a certain degree of false-positive rate in the diagnosis of ovarian cancer. For instance, it can be detected in pathological conditions such as endometriosis, uterine leiomyoma, pelvic inflammation, and ovarian tumors and physiological processes such as menstruation and pregnancy [2]. Histopathological examination is the gold standard for the diagnosis of malignant tumors, whereas it has not been widely applied in cancer diagnosis because of its invasiveness and high cost [3]. Based on the above-mentioned view, it is of great clinical significance to discover the diagnostic molecular markers with high specificity and less trauma.

In recent years, the role of miR-21 in tumor pathogenesis has been increasingly clarified, where miR-21 is involved in all known tumor biological processes, including tumorigenesis, development and metastasis [4–6]. Multiple studies have found that miR-21 is highly expressed in various malignant tumors, such as breast cancer, malignant glioma, hepatocellular carcinoma, cholangiocarcinoma, lung cancer, tongue squamous cell carcinoma, esophageal cancer, gastric cancer, colon cancer, chronic myeloid leukemia, cervical cancer, prostate cancer, and ovarian cancer [7–18]. Hence, miR-21 in the circulatory system can be adopted as a marker for cancer diagnosis [19]. There have been numerous related studies, like ones that suggest extracellular vesicle miR-21 in cerebrospinal fluid can perform as a biomarker for the diagnosis of gliomas [20]. MiR-21 is also of great significance in the diagnosis of prostate cancer [21], and the expression of miR-21 in serum and feces can be used as a potential diagnostic index of colorectal cancer [22]. A related meta-analysis also showed that exocrine miR-21 was prone to be widely applied as a general marker for cancer screening [23]. There is a high expression of miR-21, 92, 93 in the serum of patients with ovarian cancer before the increase of CA-125, indicating that it can be used as a marker for early diagnosis [24].

In order to obtain more accurate evidence to prove the relationship between the high expression of miR-21 and the diagnosis of ovarian cancer, this study conducted a comprehensive search of relevant literature and applied meta-analysis aiming to accurately assess the diagnostic value of serum miR-21 in ovarian cancer.

## Methods

We preformed this meta-analysis according to the guidelines of the preferred reporting items for systematic reviews and meta-analyses (PRISMA) [25].

### Retrieval strategy

PubMed, Embase, Web of Science, Cochrane Library, China National Knowledge Infrastructure (CNKI), and China WanFang databases were searched about the studies on the diagnostic value of miR-21 for ovarian cancer. The retrieve time was from inception to March 7, 2022. The search strategy was as follows: (“microRNA-21” OR “miRNA-21” OR “microRNA21” OR “miR-21” OR “miRNA21”) AND (“epithelial ovarian cancer” OR “ovarian cancer” OR “OC”). There was no language limitation. The titles and abstracts were checked by two researchers to identify all relevant trials, after then full text articles were scanned. The references of the relative review, and meta-analysis were also examined to identify the eligible studies. Two researchers searched each database independently, and finally cross-checked. If there were disagreements, they were resolved through discussion.

### Literature selection criteria

**Inclusion criteria**: (1) studies aimed at miR-21 in serum samples of ovarian cancer; (2) studies focused on human samples; (3) studies were on the correlation between miR-21 and diagnostic accuracy; (4) studies provided data to extract or calculate true positive (TP), false positive (FP), false negative (FN), and true negative (TN).

**Exclusion criteria:** (1) letters, case reports, reviews, conference abstracts, animal research; (2) diagnostic sensitivity and specificity could not be extracted; (3) repeated samples.

### Quality evaluation

The Quality Assessment of Diagnostic Accuracy Studies-2 (QUADAS-2) was applied for quality control of the included literature [26]. The QUADAS-2 mainly consists of 11 items, covering four aspects of patient selection, index test, reference standard, flow and timing, where the bias risk of each item should be assessed. According to the answers to the relevant typical questions in each part, answers determined by Yes (score 1), No (score − 1), or Uncertainty (score 0) were recorded. The bias risk level was judged as “low,” “high” or “moderate.” If there were disagreements between the two researchers, they resolved them by discussion.

### Data extraction

The following information upon the studies of the diagnostic value was extracted: (1) first author, year of publication, country, source of the sample, test method, number of patients, source of the control group, study duration, tumor stage, cut-off value, age; (2) sensitivity, specificity, the number of TP, FP, FN, TN. Data extraction was carried out independently by two researchers, disagreements resolved by discussion.

### Statistical analysis

Stata 15.0 statistical software (Stata Corp LLC, College Station, TX, USA) was adopted to analyze the data and output the graph of bias risk assessment of the included studies. The diagnostic threshold effect was evaluated by the Spearman correlation between the logarithm of sensitivity and the logarithm of 1-specificity and the typical “shoulder-arm shape” in summary receiver operating characteristic (SROC) curve. We adopted the bivariate mixed-effects model to combine and analyze the data in our meta-analysis. The pooled sensitivity, specificity, positive likelihood ratio (PLR), negative likelihood ratio (NLR), and diagnostic odds ratio (DOR) were calculated, and the corresponding forest plot was drawn. The area under the curve (AUC) was obtained, where the AUC value ranged from 0.5 to 1.0. When it was close to 0.5, it indicated inadequate diagnostic performance. When it was close to 1.0, it indicated sound diagnostic performance. In addition, the inconsistency index (*I*^2^) and *P* value were used to evaluate the heterogeneity among the studies [27]. Heterogeneity was judged to be significant if *I*^2^ > 50% or *P*> 0.05. The relationship between the prior probability, the likelihood ratio and the posterior test probability were evaluated by Fagan’s Nomogram plot [28]. The meta-regression and subgroup analysis were used to analyze the sources of the heterogeneity across the included studies. Several groups were conducted for meta-regression and subgroup analyses, including country (China or Egypt), consumed time of the study (<2 or ≥ 2 year), control group (health or benign), case sample size (≤80 or > 80 patients), and cut-off value (≤1.5 or > 1.5). The sensitivity analysis was used to verify the robustness of the findings. The Deeks’ funnel plot was used to assess the publication bias.

## Results

### Literature retrieval and characteristics of eligible studies

**Fig. 1:**
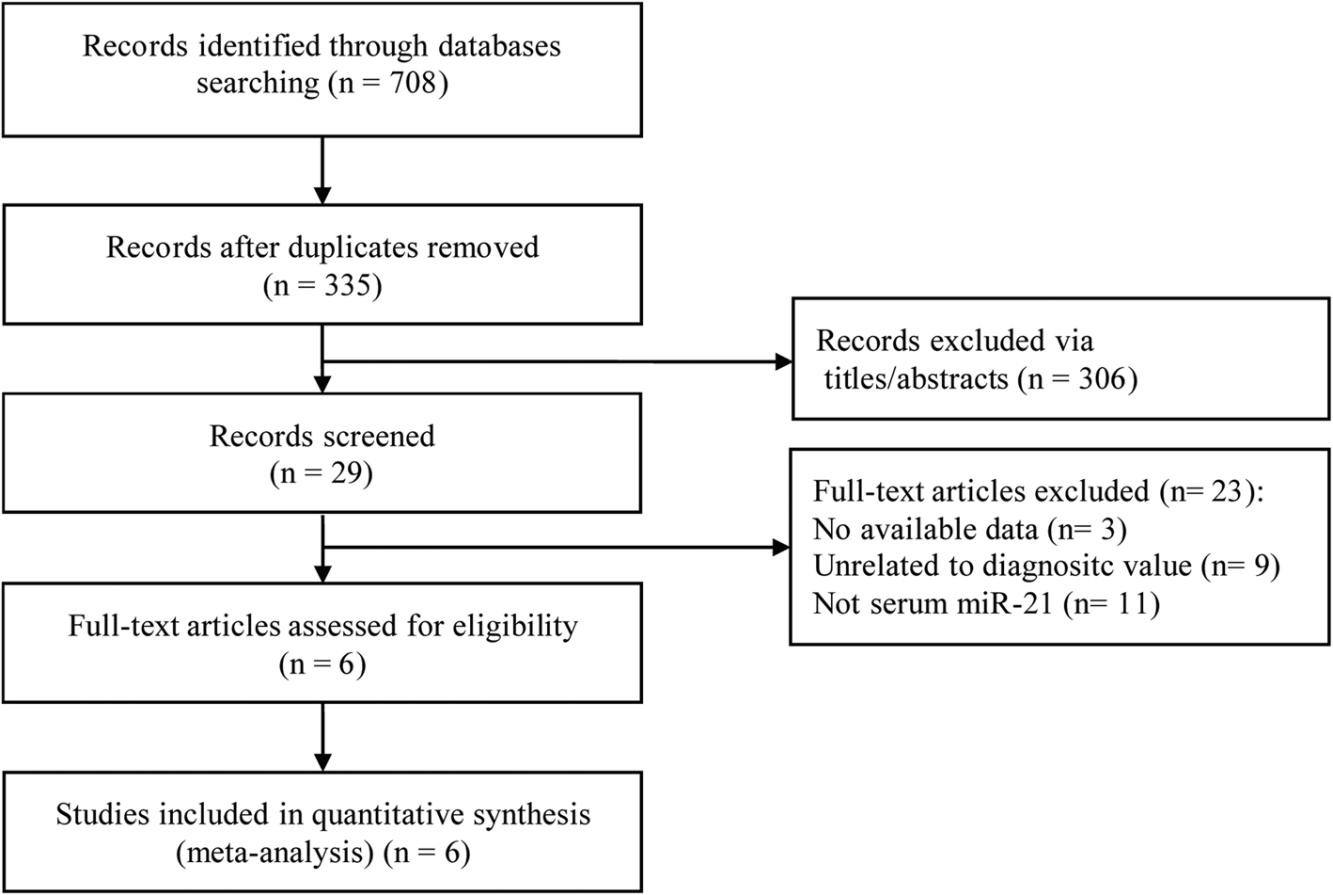
A flow diagram of the study selection process

A total of 708 articles were retrieved through the search strategy, of which the 373 repetitive articles were removed, 306 irrelevant articles were excluded through review of their titles and abstracts. After that, 23 articles were excluded. A total of 6 studies were included in this meta-analysis after checking full-text and data integrity [29–34]. A total of 1010 cases were included in the study, including the case group (*n* = 524) and the control group (*n* = 486). The specific screening flow diagram is shown in Fig. 1, and the basic characteristics and more details in the included literature are shown in Table 1.

**Table 1:** The main characteristics of the included studies in the meta-analysis

Study	Year	Country	detection method	case (control)	control (benign)	Age (median)	Time-consuming	tumor stage	Cut-off value	sensitivity	Specificity	TP	FP	FN	TN	QUADAS-2 scores
Zeng Y [29]	2016	China	RT-qPCR	40(82)	82(42)	49	1.5	I**-IV**	NR	0.675	0.7857	27	18	13	64	7
Mahmoud [30]	2018	Egypt	RT-qPCR	60(30)	30(0)	55.5	1	I, III**-IV**	1.005	0.967	0.8	58	6	2	24	9
Tang M [31]	2018	China	RT-qPCR	162(108)	108(0)	54.27	4	I**-IV**	1.46	0.842	0.705	136	32	26	76	5
Li HP [32]	2019	China	RT-qPCR	96(80)	80(80)	51.27	2	I**-IV**	1.73	0.7708	0.8125	74	15	22	65	8
You J [33]	2019	China	RT-qPCR	80(100)	100(50)	53.68	2	I**-IV**	2.13	0.788	0.92	63	8	17	92	7
Song KW [34]	2020	China	RT-qPCR	86(86)	86(0)	46.1	1	I**-IV**	1.027	0.763	0.856	66	12	20	74	9

Abbreviations: *TN* true negative, *TP* true positive, *FN* false negative, *FP* false positive, *NR* not-reported, *RT-qPCR* real-time quantitative polymerase chain reaction, *QUADAS-2* Quality Assessment of Diagnostic Accuracy Studies-2

### Quality assessment of the included studies following the QUADAS-2 criteria

**Fig. 2:**
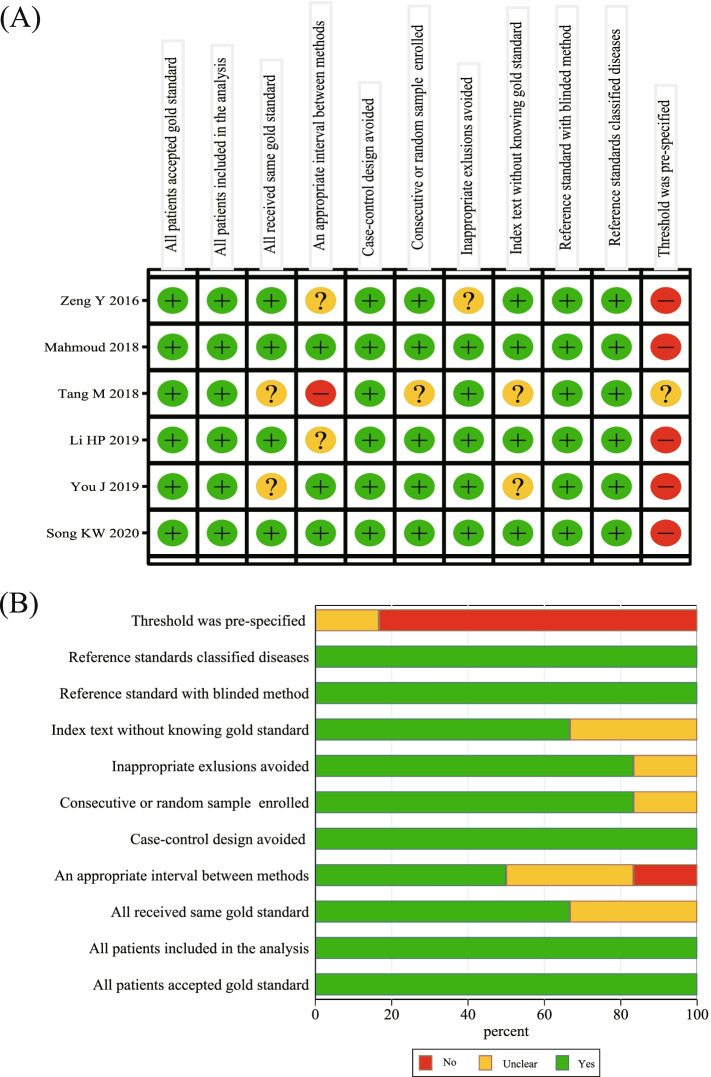
The quality of included articles according to the QUADAS-2 guidelines. **A** Risk of bias summary; **B** Risk of bias graph. QUADAS-2: Quality Assessment of Diagnostic Accuracy Studies-2

The quality of the included literature was assessed following the QUADAS-2, shown in Fig. 2A, B. The QUADAS-2 scores of the included studies ranged from 5 to 9 (Table 1). As shown in Fig. 2, the high risk of bias mainly existed in whether the threshold was pre-specified, including five studies [29, 30, 32–34]. One item was whether there was an appropriate interval between index test and reference standard, with the moderate-high bias risk accounting for 50% [29, 31, 32]. Apart from the above two items, the other nine items had low-moderate risk of bias, with the low risk of bias ranging from 66.6 to 100.0%.

### Meta-analysis results and heterogeneity analysis of diagnostic accuracy

**Fig. 3:**
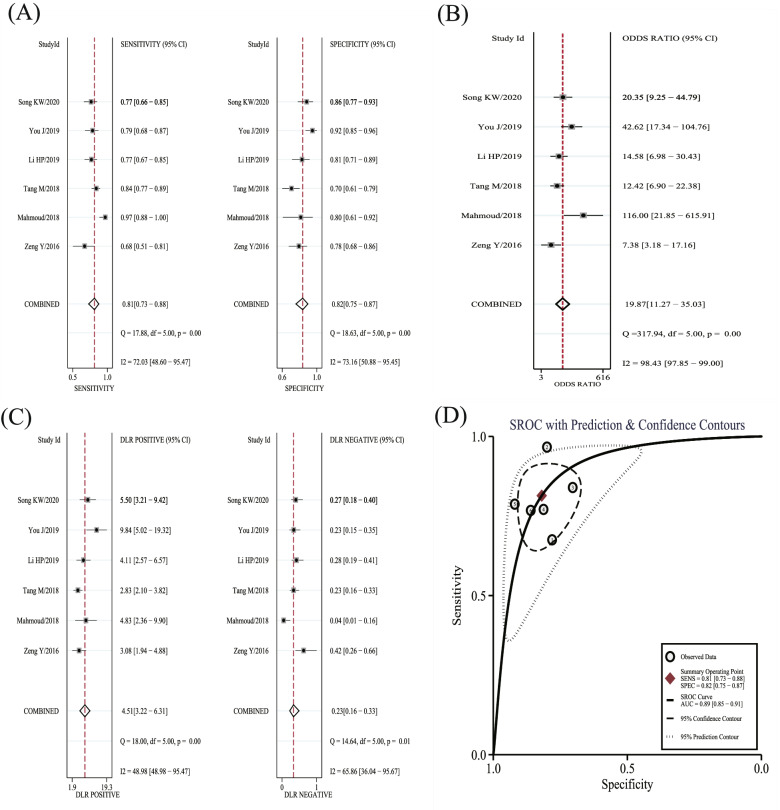
Forest plots of miR-21 for the diagnostic value of ovarian cancer. **A** sensitivity and specificity. **B** DOR. **C** PLR and NLR. **D** SROC. DOR: diagnostic odds ratio; PLR: positive likelihood ratio; NLR: negative likelihood ratio; SROC: summary receiver operating characteristic

**Fig. 4:**
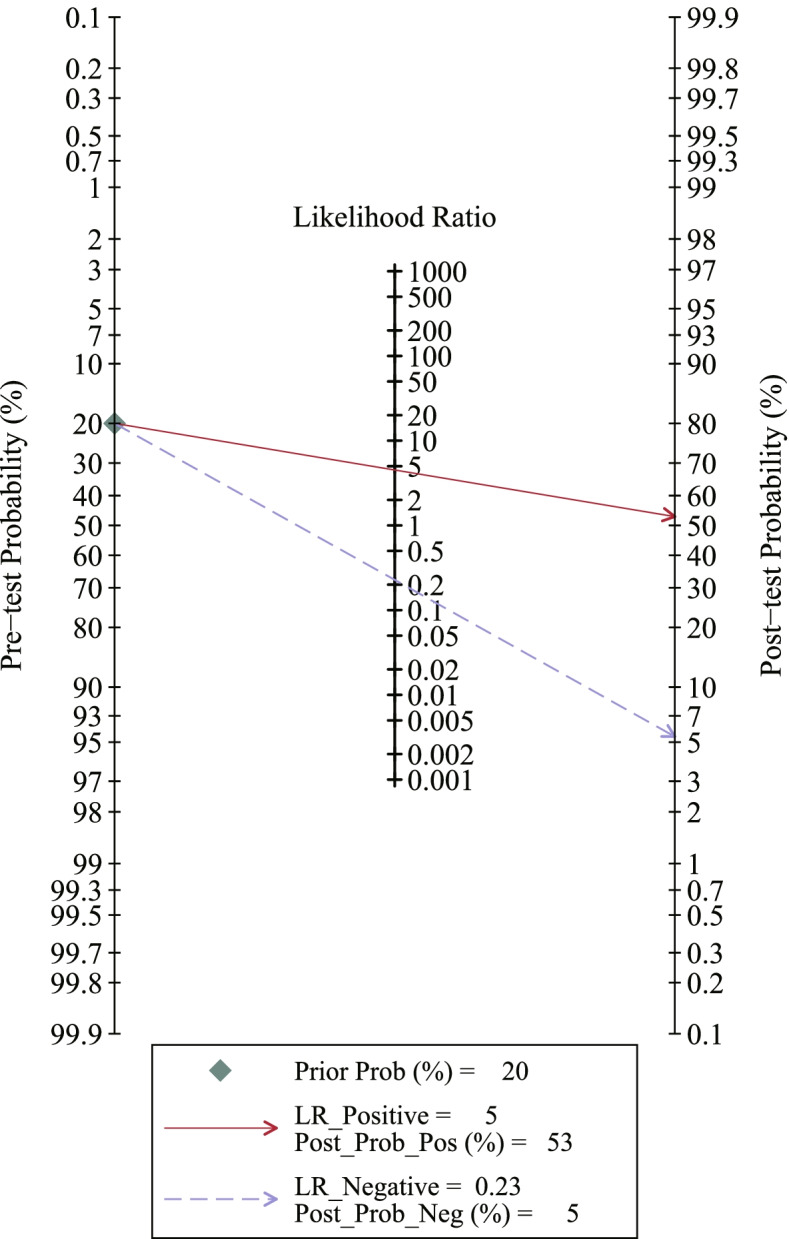
Fagan’s plot of PLR and NLR to evaluate the clinical utility of miR-21 in the diagnosis of ovarian cancer. PLR: positive likelihood ratio; NLR: negative likelihood ratio

The forest plots and SROC of the diagnostic accuracy of miR-21 are shown in Fig. 3(A, B, C, D). The SROC curve (Fig. 3D) did not show a typical "shoulder-arm shape" and Spearman correlation was 0.143 (*P* > 0.05), indicating no threshold effect among the diagnostic test. The DOR (*P* < 0.001, *I*^2^ = 98.43%) (Fig. 3B), sensitivity (*P* < 0.001, *I*^2^ = 72.03), specificity (*P* < 0.001, *I*^2^ = 73.16%) (Fig. 3A), PLR (*P* < 0.001, *I*^2^ = 48.98%), and NLR (*P* < 0.001, *I*^2^ = 65.86%) (Fig. 3C) showed significant heterogeneity in the pooled analysis. The results of the bivariate mixed-effects model showed that, the pooled sensitivity, specificity, PLR, NLR, and DOR were 0.81 (95%CI: 0.73–0.88), 0.82 (95%CI: 0.75–0.87), 4.51 (95%CI: 3.22–6.31), 0.23 (95%CI: 0.16–0.33), and 19.87 (95%CI: 11.27–35.03), respectively. Besides, the AUC was 0.89 (95%CI: 0.85–0.91). The Fagan’s Nomogram showed that, if the pre-test probability ratio was 20%, the post-test probability of PLR was 53%, while the post-test probability of NLR was 5% (Fig. 4), which indicated that miR-21 has a good value in the diagnosis of ovarian cancer.

### Meta-regression and subgroup analyses to explore the sources of the heterogeneity across the included studies

**Fig. 5:**
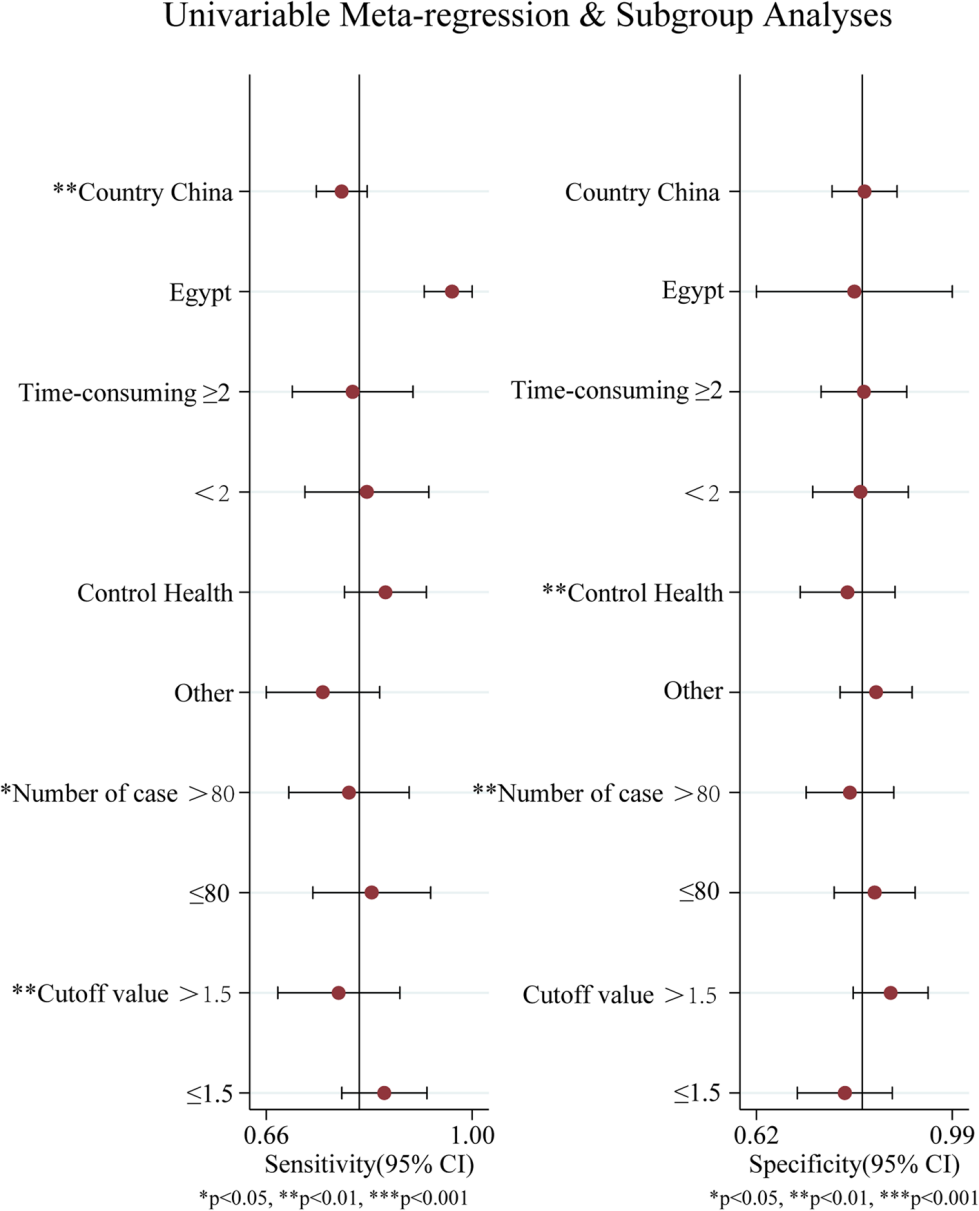
Mate-regression and subgroup analyses

The results showed that the diagnostic value of miR-21 varied in different races, sample sizes and cut-off values (*P* < 0.05), which all may be the source of heterogeneity (Fig. 5). The combined sensitivity of the Egyptian group was higher than that of the Chinese group, the combined sensitivity of sample size ≤80 group was higher than that of sample size > 80 group, and the combined sensitivity of cut-off value ≤1.5 group was higher than that of cut-off value > 1.5 group, which was statistically significant. Subgroup analysis showed the diagnostic value of that both the Egyptian group and the control group were healthy population group, sample size ≤80 group and cut-off value ≤1.5 were higher than that of the corresponding group.

### Sensitivity analysis to verify the robustness of the findings

**Fig. 6:**
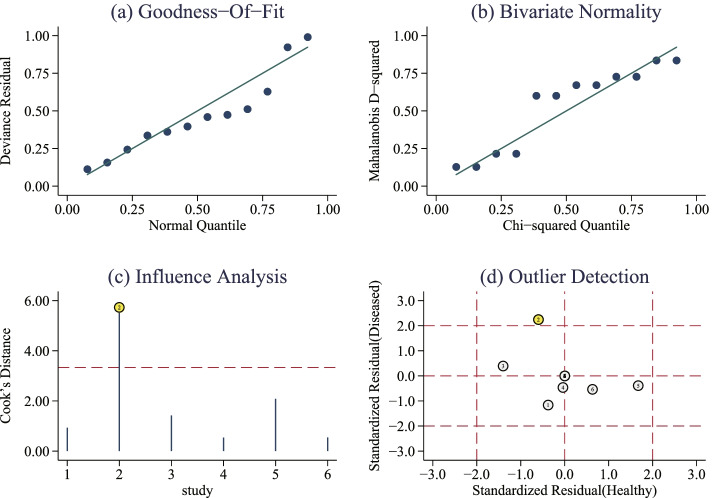
The results of sensitivity analysis. **A** Goodness-of-fit. **B** Bivariate normality. **C** Influence analysis. **D** Outlier detection

The sensitivity analysis showed that goodness-of-fit and bivariate normality fit favorably (Fig. 6A, B). Influence analysis found that there was a document weighted (Fig. 6C) [30], which might be the source of heterogeneity indicated by abnormal value detection (Fig. 6D). After excluding the abnormal study, the sensitivity decreased slightly from 0.81 to 0.79, AUC from 0.89 to 0.84, and DOR from 19.87 to 17.00.

### Detection of the publication bias

**Fig. 7:**
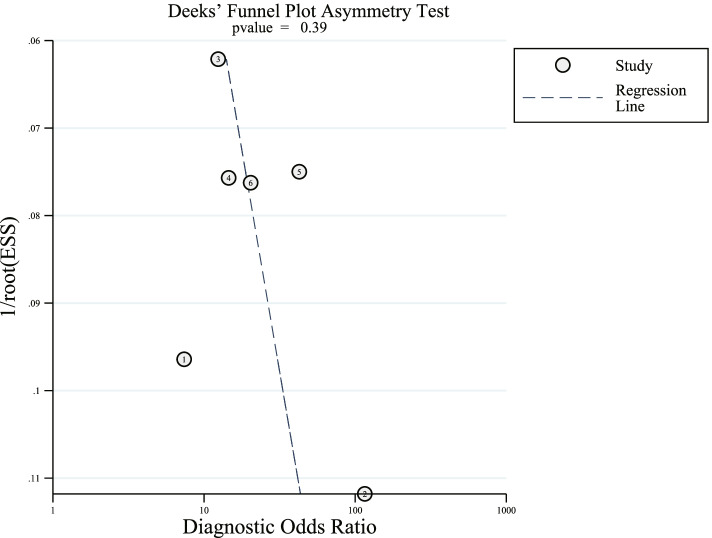
The Deeks’ funnel plot for assessing the publication bias

The Deeks’ funnel plot shows that the *p*-value is 0.39, which indicates there is no publication bias in this meta-analysis (Fig. 7).

## Discussion

Histopathological examination is the gold standard for the diagnosis of malignant tumors, whereas it has not been widely applied in cancer diagnosis because of its invasiveness and high cost [3]. At present, a variety of biomarkers such as carcinoembryonic antigen (CEA), cancer antigen 125 (CA125), cancer antigen 15–3 (CA15–3), alpha-fetoprotein (AFP) were applied in the clinical diagnosis of tumors, whose sensitivity and specificity was low [35, 36]. Disorders of miR-21 regulation occur in a variety of cancers, such as breast cancer, colon cancer, lung cancer, prostate cancer, pancreatic cancer, gastric cancer, ovarian cancer, esophageal cancer, and liver cancer, which were used as a qualitative and quantitative marker for potential cancer detection, as well as potential targets for treatment [37]. Certain miRNAs, such as miR-200 family, let-7 family, miR-21, miR-214 and miR-100, have the potential diagnostic and prognostic value in ovarian cancer through the special signaling pathway [38]. MiR-21 regulates the proliferation and apoptosis of ovarian cancer cells through the PTEN/PI3K/AKT pathway [39] and the jagged-1 pathway [40], regulating cell invasion, migration and colony formation by the miR-21/Wnt/CD44v6 pathway [41]. The 3-year survival rate with a low expression of serum miR-21 was higher than that with a high miR-21 expression of patients with ovarian cancer [34]. High-level serum miR-21 expression (Overall survival: HR = 2.327, *P* = 0.019) was an unfavorable prognostic factor in the multivariate cox-regression model [42]. Besides, up-regulated miR-21 were observed in tumor tissue, positively correlated with tumor grade and tumor stage in ovarian cancer [43]. Multiple studies have shown that plasma miR-21 is highly expressed in ovarian cancer and is a potential new molecular marker for the diagnosis of ovarian cancer [44–47]. The expression profile of miRNAs in tissue and serum of patients with ovarian cancer is related to tumorigenesis and prognosis [48]. However, the above-mentioned detection technique of tissue samples is complex and traumatic, while the detection of serum miRNAs is superior in its convenient sampling, simple operation, less trauma, low cost and easy promotion. Whether miR-21 can be used as a diagnostic molecular marker for ovarian cancer has spectacular clinical significance.

The pooled sensitivity and specificity of the six studies were 0.81 and 0.82, respectively, which suggested that miR-21 has sound sensitivity and specificity in the diagnosis of ovarian cancer. The PLR and NLR were 4.51 and 0.23, respectively, indicating that the possibility of correct judgment of positive in diagnostic test was 4.51 times higher than that of misjudgment, while the possibility of misjudging negative was 23% of that of correct judgment. It is reported that the PLR should be more than 10 and the NLR should be less than 0.1 to ensure convincing accuracy [49], which suggests that miR-21 has limited application for diagnosing ovarian cancer. The value of DOR ranges from 0 to infinity, where a higher value means better cancer diagnosis [50]. And the DOR value of this study was 19.87, suggesting that miR-21 can be used as a biomarker for the diagnosis of ovarian cancer. AUC is an index to evaluate the overall effectiveness of the diagnostic experiment, which is considered to have the accurate diagnostic ability when its value is between 0.93 and 0.96, while it is also effective when its value is between 0.75 and 0.92 [51, 52]. The AUC of the pooled ROC curve in this study was 0.89, indicating that miR-21 has favorable diagnostic accuracy. Considering all the indicators have more advantages than individual indicators, miR-21 has significant diagnostic value for ovarian cancer. The researchers collected 15 articles to analyze the diagnostic value of miR-21 in digestive system cancer. The results showed that the pooled sensitivity and specificity of liver cancer, bile duct cancer, pancreatic cancer, gastric cancer, esophageal cancer, and colorectal cancer were 0.76 (95%CI: 0.70–0.82), 0.84 (95%CI: 0.78–0.89), and the DOR value was 17.15, with AUC 0.87 [53]. The Meta-analysis also showed that miR-21 was a potential molecular marker with high sensitivity and specificity in early breast cancer, whose pooled sensitivity, specificity, and DOR were 0.79 (95%CI: 0.66–0.87), 0.85 (95%CI: 0.75–0.91), 19.46 (95%CI: 8.74–43.30), respectively, with AUC 0.89. However, another report showed that, the pooled sensitivity and specificity, PLR, NLR and DOR of miR-21 in the diagnosis of breast cancer were 0.72 (95%CI: 0.69–0.75), 0.80 (95%CI: 0.77–0.83), 3.37 (95%CI: 2.24–5.07), 0.30 (95%CI: 0.19–0.50), 11.79 (95%CI: 5.23–26.57), respectively, with AUC 0.8517 [54, 55]. MiR-21 and miR-210 can also be used as diagnostic tools for non-small cell lung cancer, especially in Caucasians and non-smokers [56]. MiR-21 has better diagnostic value than miR-125 and miR-222 in gliomas, of which the pooled sensitivity, specificity, PLR, NLR and DOR were 0.84 (95%CI: 0.76–0.90), 0.87 (95%CI: 0.63–0.97), 6.6 (95%CI: 1.9–23.1), 0.18 (95%CI 0.11–0.31), 36 (95%CI: 7–187), respectively, with AUC 0.88 [57]. Therefore, this meta-analysis shows that the serum miR-21 has a good diagnostic value in ovarian cancer, suggesting that miR-21 could be a reference biomarker for the diagnostic performance in patients with ovarian cancer.

This study has found that the heterogeneity of the pooled effect is not caused by the threshold effect, which means heterogeneity results from other factors. It was meta-regression analysis that discovered such factors as different sample sizes of ovarian cancer patients, different national populations and different cut-off values were probably important sources of heterogeneity. The reason might be that the demographic characteristics varied among different regions, and so did the detection rate of positive results under different sample sizes and different cut-off values. The subgroup analysis showed that the diagnostic value of Egypt group, control group, sample size ≤80 group and cut-off value ≤1.5 group were higher than that of the corresponding groups. The result is significant in guiding further data collection of the follow-up research, and more than that, it provides a subgroup basis for the subsequent subgroup analysis. The results of sensitivity analysis show that the results of this study are stable. No significant publication bias was found in this meta-analysis.

Indeed, this meta-analysis was limited by the following factors. Firstly, the time when miR-21 was used as a molecular marker of tumor was short, with relatively a few samples included. Accordingly, more studies are needed with larger sample size. Secondly, early detection of ovarian cancer means a lot to clinically significance. In this study only one article discussed the diagnostic value of miR-21 in early ovarian cancer [32], two articles [29, 31] mentioned that there are differences in miR-21 levels between early and late patients. More researches are needed to study different clinical stages, tissue differentiation, and miR-21 diagnosis in lymph node metastasis of ovarian cancer. Thirdly, the included literature showed that miR-21 combined with other molecular markers or diagnostic methods could improve the sensitivity of diagnosis, such as miR-203 [29], PDCD4 [30], miR-200b [31], HE, Finkler ultrasound score [32], CA125 [33], miR-26b [58], We couldn’t analyze the combined effect due to the limited information, which is significant in guiding further research. Fourthly, the relationship between miR-21 and the prognosis of ovarian cancer is also very important but pooled analysis of the prognostic value of miR-21 in ovarian cancer couldn’t be performed in this study due to the limited information of the current literature.

In conclusion, serum miR-21 has a good diagnostic value in ovarian cancer, which we can consider combining with other biomarkers or other diagnostic methods to improve the diagnostic accuracy of patients with ovarian cancer. Given the heterogeneity of the research, more samples with larger sizes are still needed to verify the findings prospectively in the future.

## Data Availability

The datasets used and/or analysed during the current study are available from the corresponding author on reasonable request.

## Abbreviations

DOR: Diagnostic odds ratio
PLR: Positive likelihood ratio
NLR: Negative likelihood ratio
AUC: Area under the curve
EOC: Epithelial ovarian cancer
CA125: Carbohydrate antigen 125
QUADAS-2: Quality Assessment of Diagnostic Accuracy Studies-2
CEA: Carcinoembryonic antigen
CA15–3: Cancer antigen 15–3
AFP: Alpha-fetoprotein
SROC: Summary receiver operating characteristic
HRs: Hazard ratios
OS: Overall survival
FIGO: Federation International of Gynecology and Obstetrics
